# Applications of Reverse Osmosis and Nanofiltration Membrane Process in Wine and Beer Industry

**DOI:** 10.3390/membranes15050140

**Published:** 2025-05-02

**Authors:** Yogesh Kumar, Atul Khalangre, Rajat Suhag, Alfredo Cassano

**Affiliations:** 1 Department of Agricultural and Food Sciences, University of Bologna, Piazza Goidanich 60, 47521 Cesena, Italy; 2 Interdepartmental Centre for Agri-Food Industrial Research, University of Bologna, Via Q. Bucci 336, 47522 Cesena, Italy; atul.khalangre2@unibo.it; 3 Faculty of Agricultural, Environmental and Food Sciences, Free University of Bolzano, Piazza Università 1, 39100 Bolzano, Italy; rajat.suhag@unibz.it; 4 Institute on Membrane Technology, ITM-CNR, Via P. Bucci, 17/C, 87036 Rende, Italy; a.cassano@itm.cnr.it

**Keywords:** membranes, reverse osmosis, nanofiltration, dealcoholization, wine, beer

## Abstract

Reverse osmosis (RO) and nanofiltration (NF) membranes are traditionally employed in wine and beer production for concentration, clarification, and stabilization. Their applications now extend to dealcoholization, addressing rising demand for low-alcohol beverages. RO/NF selectively reduce ethanol while retaining volatile aromas and non-volatile flavors, outperforming thermal methods that degrade sensory profiles. This review examines RO/NF roles in alcohol adjustment, sugar modification, and by-product recovery, emphasizing mechanisms and efficiency. Operational challenges such as membrane fouling (polysaccharides, polyphenols), selectivity–permeation flux trade-offs, and energy costs are assessed. By balancing tradition with innovation, RO/NF technologies offer transformative potential for meeting health and sustainability goals in beverage industries. However, gaps in standardization, sensory consistency, and cost-effectiveness necessitate targeted research to optimize industrial adoption and consumer acceptance.

## 1. Introduction

The wine and beer industries, deeply rooted in cultural traditions and economic significance, are navigating an era of unprecedented transformation. As global consumer preferences shift toward healthier lifestyles and sustainability, producers face dual challenges: meeting demand for innovative products like low-alcohol and alcohol-free beverages while adhering to stringent environmental regulations and resource-efficiency goals [1,2]. Traditional methods for adjusting alcohol content, concentrating juices, or treating wastewater—such as thermal evaporation, vacuum distillation, and chemical treatments—often compromise product quality, consume excessive energy, or generate environmental loads [3,4]. In this context, membrane-based separation technologies, particularly, RO and NF, have emerged as versatile alternatives that align with modern industrial priorities of precision, sustainability, and cost-effectiveness.

**Figure 1:**
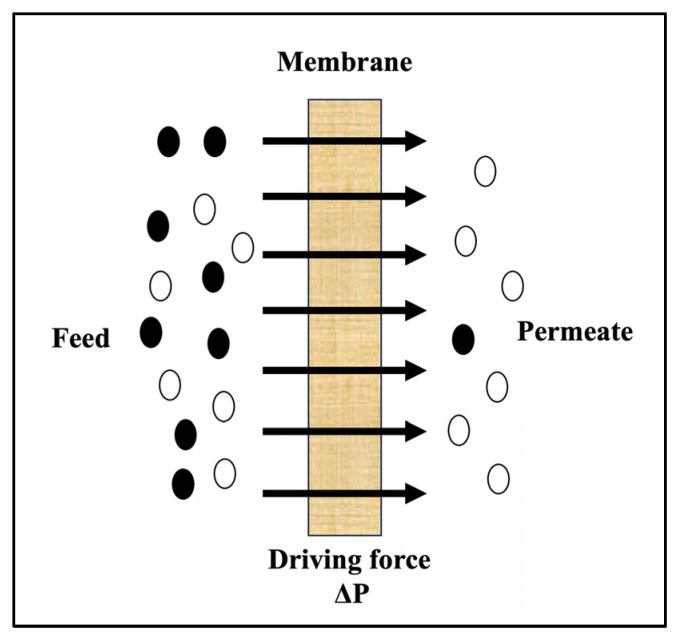
Principle of RO and NF membranes. ∆P: Pressure gradient. Black circles represent larger molecules (e.g., polyphenols, other macromolecules) retained by the membrane, while white circles represent smaller molecules (e.g., water, ethanol) that permeate through the membrane.

RO and NF processes operate on the principle of selective solute separation through semi-permeable membranes under applied pressure (Figure 1) [5,6]. While RO membranes effectively retain dissolved solutes, including salts, sugars, and small organic molecules, NF membranes offer a middle ground, allowing partial permeation of monovalent ions (e.g., sodium, chloride) while retaining larger molecules like sugars and polyphenols (Table 1) [7]. This unique selectivity makes NF ideal for applications requiring fine-tuned separation, such as adjusting sugar content in wine musts or preserving hop-derived flavor compounds in beer [8,9]. RO, with its tighter membrane structure, excels in scenarios demanding high purity, such as dealcoholization or wastewater reclamation [10]. Both technologies minimize thermal and chemical stress on products, preserving delicate sensory profiles, a critical advantage in industries where aroma, flavor, and mouthfeel define market success [11,12,13].

In winemaking, RO and NF are revolutionizing practices such as must concentration to enhance wine body without over-reliance on sugar additives, dealcoholization to cater to health-conscious consumers, and recovery of valuable by-products like tartaric acid or phenolic compounds from waste streams [15,16,17,18]. Simultaneously, these membranes enable wineries to treat and reuse wastewater, reducing freshwater consumption and environmental discharge [19,20,21]. In brewing, RO and NF facilitate the production of alcohol-free and low-alcohol beers—a rapidly growing market segment—by selectively removing ethanol while retaining volatile aroma compounds. They also address quality control challenges, such as eliminating off-flavors (e.g., diacetyl, sulfites) and stabilizing hop bitterness during storage. Moreover, breweries are adopting these technologies for water reuse, a critical step toward achieving circular economy goals in water-stressed regions [8,22,23].

Despite their promise, the integration of RO and NF into these industries is not without hurdles. Membrane fouling caused by organic matter, colloids, or scaling remains a persistent operational challenge, necessitating rigorous cleaning protocols and increasing downtime [24,25,26]. Energy consumption, though lower than thermal methods, still impacts operational costs, particularly in large-scale applications [10,26]. There are also concerns about potential sensory alterations: while RO and NF generally preserve key flavor components better than traditional methods, subtle changes in acidity, bitterness, or aromatic complexity may require careful optimization [7,11]. Regulatory frameworks further complicate adoption, as labeling laws for “alcohol-free” beverages vary globally, and consumer perceptions of membrane-processed products remain untested in some markets.

This review explores the role of RO and NF in addressing critical needs across wine and beer production, evaluates their benefits and limitations, and highlights innovations that enhance their feasibility. By synthesizing current research and industrial practices, this paper aims to provide a comprehensive resource for stakeholders seeking sustainable, efficient solutions in alignment with modern industry standards.

## 2. Fundamentals of RO and NF Membrane Processes

RO and NF are pressure-driven membrane processes that separate two solutions with different concentrations using a semi-permeable membrane [27]. These membranes are typically composed of polyamide thin-film composites (TFCs), especially phenylene diamine polyamide used for RO and piperazine polyamide for NF, which exhibit excellent separation performance and chemical resistance. Membrane configurations include spiral-wound, plate-and-frame, hollow fiber, tubular, capillary, and flat-sheet designs, which can be manufactured from polymeric or ceramic materials as per the requirements. While these configurations influence operational efficiency, packing density, and application suitability, the fundamental separation performance of RO and NF membranes is governed by their material composition and pore structure rather than module design. Furthermore, RO membranes have pore sizes below 0.001 μm and molecular weight cutoffs (MWCOs) under 100 Da, enabling them to effectively retain solutes such as monovalent salts and small organic molecules. In contrast, NF membranes have slightly larger pores (0.001–0.01 μm) and MWCOs ranging from 200 to 1000 Daltons. As a result, NF membranes allow for greater permeability; however, they exhibit relatively lower rejection rates for certain compounds compared to RO membranes. This structural difference grants NF membranes greater permeability but relatively lower rejection rates for certain compounds compared to RO membranes [28]. Moreover, operating conditions vary depending on the application and membrane type. Traditional RO processes typically require high pressures ranging from 10 to 100 bar, though advanced low-energy RO systems can operate below 10 bar. NF generally operates at lower pressures between 2 and 40 bar. Temperature tolerances for standard membranes are typically 45–50 °C, but specialized high-temperature-resistant NF membranes can withstand temperatures exceeding 50 °C [10,14,27,28,29].

The volume reduction factor for these processes can be determined using Equation (1)

(1) $$VRF=\frac{V_{f}}{V_{r}}$$

where *V_f_* is the initial feed volume (L), and *V_r_* is the retentate volume (L).

The permeate flux and permeability can be calculated using Equations (2) and (3), respectively [29,30].

(2) $$J=\frac{V}{A\times t}$$

(3) $$L_{p}=\frac{J}{P}$$

where

1. *J* (L/m^2^h) = permeate flux;
2. *Lp* (L/m^2^hbar) = permeability;
3. *P* (bar) = applied pressure;
4. *V* (L) = collected permeate volume during the process;
5. *A* (m^2^) = effective membrane area;
6. *t* (h) = operating time.

Furthermore, the fouling index (FI) can be determined using Equation (4) [31,32].

(4) $$FI\;(\%)=\frac{L_{p1}}{L_{p0}}$$

where L*p*_1_ and *Lp*_0_ are the pure water permeability (L/m^2^hbar) before and after processing, respectively.

The overall rejection (R) of selected membranes towards various components during the process can be calculated by using Equation (5) [33].

(5) $$R\;\left(\%\right)=\left(1-\frac{C_{pi}}{C_{ri}}\right)\times 100$$

where

1. *Cpi* = concentration of i compound in the permeate;
2. *Cri* = concentration of i compound in the retentate side.

## 3. Wine Industry

RO and NF membrane techniques offer promising applications in the wine industry for enhancing quality and sustainability, although their use is currently limited to specific processes and contexts. These membranes are commonly utilized to clear wines by eliminating haze-causing proteins, stabilizing compositions without heat, and reducing alcohol content by selectively removing ethanol. They also concentrate grape musts, increasing sugar and taste intensity, and assuring minimum flavor influence. Furthermore, producing alcohol-free wines, recovering important by-products like polyphenols, and combining with other processes for energy-efficient operations are examples of recent developments, although issues like membrane fouling and expenses still need to be addressed.

### 3.1. Concentration of Must and Sugar Adjustment

The grapes may have low sugar content, possibly due to imperfect ripening caused by unfavorable weather conditions, resulting in wine of inferior quality and insufficient alcohol content to meet legal requirements [34]. Thus, to increase the sugar concentration in the must, water is removed to concentrate it, and the water extraction process is usually performed using NF and RO membranes [35,36]. Bianchi et al. [34] concentrated the grapes musts of Trebbiano, Verdeca, Black Bombino, and White Bombino varieties using RO and NF membranes. Pati et al. [37] used RO and NF membranes to improve the quality of grape must. The results indicated that the concentrated must had higher soluble solids, titratable acidity, and color intensity compared to untreated must. Meanwhile, the wine produced from RO- and NF-concentrated must exhibited higher titratable acidity, alcohol concentration, dry extract, color intensity, and total phenols, while no significant changes were observed in pH and volatile acidity compared to the wine produced from untreated must. Similarly, Santos et al. [38] observed an increase in the sugar content of model musts concentrated by different types of NF membranes (ET NA 0.1 PP, NFT50, NF90, NF200, NF270, and CA27-87.5). In a study, Versari et al. [39] evaluated the efficiency of NF membranes in concentrating grape must and found that these NF membranes exhibited rejection coefficients ranging from 77% to 97% for sugars. Furthermore, the combination of RO (60 bar, 20 °C) and NF (70 bar, 40 °C) membranes efficiently concentrated grape must, increasing the sugar content to around 45°Brix [40]. However, volatile compounds, especially esters, higher alcohols, and monoterpenes, can be partially lost during process depending on membrane selectivity and operating conditions [41]. In a recent study, Yogesh et al. [7] concentrated commercial white wine (Tavernello) using nanofiltration (NF-DK) and reverse osmosis (RO-SG) membranes at a weight reduction factor (WRF) of 4. Their findings revealed that the NF-DK membrane achieved significantly higher retention of reducing sugars (89%) compared to the RO-SG membrane (83.4%). However, the RO-SG membrane demonstrated superior performance in retaining other key compounds: total esters (71.5%), total volatile alcohols (75.8%), total acids (50%), and total terpenes (100%). Conversely, the NF-DK membrane showed greater efficacy in retaining total carbonyl compounds (66.7%).

### 3.2. Dealcoholization

RO and NF are employed to reduce alcohol content in wine by selectively removing ethanol and water, followed by blending the treated wine with the original wine to achieve the desired alcohol level. Various factors have contributed to the rising alcohol content in wine, including global warming and quality policies implemented by professionals in the wine industry. These factors and practices have led to high sugar concentrations in grapes, resulting in unbalanced wines [3,14]. Additionally, consumer demand for low-alcohol, reduced-alcohol, and dealcoholized wines has increased in recent years due to religious beliefs, health concerns, personal preferences, and a growing shift toward non-alcoholic alternatives [42,43]. To address this issue, NF and RO are widely used to reduce or adjust the ethanol content in wine. Catarino et al. [44] investigated the effectiveness of (RO) and (NF) membranes in ethanol removal from red wine. The study evaluated one RO membrane (CA995PE) and four NF membranes (NF99 HF, NF99, NF97, and YMHLSP1905). Among the NF membranes, YMHLSP1905 exhibited the lowest ethanol rejection (6.7%), whereas across all tested membranes, including RO, CA995PE demonstrated the lowest ethanol rejection (2.5%). In terms of permeate flux, NF97 (1.48 kg m^−2^ s^−1^) and CA995PE (1.41 kg m^−2^ s^−1^) showed the lowest values, while NF99 HF exhibited the highest flux (7.10 kg m^−2^ s^−1^) after 150 min of operation. Furthermore, Gil et al. [45] used an RO membrane to reduce the alcohol content of Cabernet Sauvignon red wine by approximately one degree (−1%) and two degrees (−2%). Ivic’ et al. [15] produced low-alcohol red wine (Cabernet Sauvignon) using NF membranes. The process was carried out with Alfa Laval NF M20 flat-sheet polyamide membranes, both with and without cooling, at four different pressures (2.5, 3.5, 4.5, and 5.5 MPa). Each experimental run began with 3 L of wine at 15 °C and was concentrated to yield 1.3 L of retentate and 1.7 L of permeate. To compare the composition of the retentate with the original wine, the separated permeate volume was replaced with distilled water before analysis, restoring the initial wine volume to 3 L. The results indicated that increasing the pressure from 2.5 to 5.5 MPa led to an increase in the fouling index from 28.59% to 31.45%. The produced low-alcohol wines had an alcohol content ranging from 5.65% to 6.30% *v*/*v*. In the low-alcohol wine, total acidity, volatile acidity, malic acid, lactic acid, citric acid, and pH significantly decreased, while no significant change was observed in tartaric acid compared to the original wine. Higher pressure and retentate cooling were more favorable for total volatile retention than lower pressure and higher temperature. Finally, total acidity, total volatile alcohol, total esters, and total volatile phenols were reduced by 58–77%, 59.7–79.5%, 48–60%, and 45–60%, respectively. In a recent study, Yogesh et al. [29] compared three NF membranes (TS 40, NF99, HL) and one RO membrane (RO-SE) to reduce ethanol from SanCrispino commercial white wine. The results indicated that the HL and NF99 membranes were more effective in reducing the alcohol content in wine, with ethanol rejection rates of 5.14% and 5.46%, respectively. Additionally, the NF99 and HL membranes exhibited the highest rejection rates for reducing sugars (>90%), glucose (>80%), fructose (>88%), citric acid (>88%), and tartaric acid (>89%) in dealcoholized wine.

Sam et al. [11] used an industrial-scale RO plant to produce non-alcoholic wine from Chardonnay (13.4% *v*/*v* ethanol), Pinot Noir rosé (12.2% *v*/*v* ethanol), and Merlot (13.9% *v*/*v* ethanol), dealcoholizing all wines to 0.7% *v*/*v* ethanol. The results indicated that reducing sugars and color intensity significantly increased, while no significant change was observed in density. The findings demonstrated that RO-treated wines experienced overall ester concentration losses of 92%, 81%, and 87% in white, rosé, and red wines, respectively. Total volatile alcohol content decreased by 75%, 58%, and 68% in red, rosé, and white wines, respectively. The total organic acid concentration was reduced by 76%, 91%, and 73% in red, rosé, and white wines, respectively. Furthermore, the loss of volatile compounds significantly affects the sensory profile of wine. In rosé wine, RO-treated samples exhibited a noticeable decline in aroma intensity, fruity and floral notes, and red fruit attributes, while also showing a reduction in body, suggesting diminished aromatic complexity and mouthfeel following dealcoholization. In red wine, RO treatment similarly resulted in higher color intensity but lower scores for aroma intensity, red fruit character, and body, indicating a clear reduction in the retention of phenolic and volatile compounds, which are essential for the varietal identity and structural integrity of red wines. Notably, astringency and bitterness were also somewhat reduced, possibly reflecting the partial removal of polyphenols or tannin-associated compounds. In white wine, RO led to significant declines in aroma intensity, fruity and floral perception, and body, along with a modest reduction in sweetness, contributing to a flatter sensory experience. Regarding overall acceptability, no significant differences were observed between the original and RO-dealcoholized white and rosé wines, whereas the red wine received lower scores.

Furthermore, Pham et al. [46] integrated an RO membrane with evaporative perstraction to lower the alcohol content by 1.8–2.5% *v*/*v* in Cabernet Sauvignon wines. The results indicated that ethanol removal led to a significant increase in density, with no impact on pH, total acidity, or volatile acidity. However, it resulted in increased concentrations of glycerol, succinic acid, and lactic acid. Additionally, total volatile compounds increased by 5–20%. The performance of a membrane depends on many factors, such as its thickness; porosity; the structure of its top layer (whether porous or dense); geometric configuration; and material properties like glass transition temperature, composition, hydrophobicity/hydrophilicity, and surface charge [11,47,48,49]. Additionally, it depends on operating conditions such as pressure, temperature, flow speed, etc.

### 3.3. Recovery of Valuable By-Products

RO and NF are used to recover valuable by-products from winery waste streams, such as polyphenols, tartaric acid, and ethanol, for use in other industries. Quirós et al. [20] explored NF and RO membranes for polyphenol recovery from winery waste streams through aqueous-based processing. The flat-sheet membranes (NF270, NF90, and Duracid as NF membranes, and BW30LE as RO membrane) were used. The results indicated that the NF90 membrane achieved 91% total polyphenol content (TPC) rejection at 7 bar and a flux of 31.5 L m^−2^ h^−1^, outperforming the Duracid membrane, which exhibited 92% TPC rejection at 10 bar and 14.0 L m^−2^ h^−1^. Subsequent RO treatment further improved purification, achieving >99.9% TPC rejection. Similarly, Kontogiannopoulos et al. [50] reported 98% polyphenol retention using an NF90 membrane with a 12.6 cm^2^ filtration area for acidified wine lees (pH 3).

### 3.4. Wastewater Treatment

The treatment of winery wastewater using NF and RO membranes presents a sustainable approach to managing high organic loads and seasonal variability while facilitating resource recovery [51]. Winery effluents, characterized by fluctuating chemical oxygen demand (COD) levels (1–4 g/L) and polyphenol-rich content, pose challenges for conventional treatment methods. Membrane bioreactors (MBRs) integrated with NF/RO systems have demonstrated exceptional performance, achieving >95% COD removal and enabling water reuse [19,52]. Natraj et al. [53] treated distillery wastewater using both RO and NF membranes. The experiments were conducted using a hybrid NF-RO pilot plant, where the NF permeate was used as the feed for the RO system, with feed pressure varied from 0 to 70 bar. The results indicated that the NF membrane effectively removed the color, transforming the wastewater from dark brown to colorless. The pH increased from 3 to 6.5, while total dissolved solids (TDS) were reduced from 51,500 to 9050 mg/L. Chemical oxygen demand (COD) decreased from 100,000 to 2900 mg/L, the potassium concentration dropped from 2050 to 1740 mg/L, and the chloride concentration reduced from 4900 to 2650 mg/L compared to untreated distillery wastewater. The NF permeate was then used as feed for the RO system, and increasing the feed pressure significantly reduced the concentration of these compounds. At a pressure of 70 bar, the RO membrane exhibited high rejection rates: TDS (99.80%), COD (99.90%), potassium (99.99%), and chloride (83%). Ortega et al. [54] mentioned that pH has a significant impact on membrane processes; phenol rejection ranged from 95 to 15% when the pH was changed from 3 to 9. The influence of pH, particularly in systems with low MWCO membranes, is due to the amplitude of electrostatic interactions (far stronger) between molecules and membranes in relation to pressure [55]. Furthermore, Loannou et al. [56] found that RO is a promising technique for the treatment of winery wastewater with an initial COD of 5350 mg/L. The RO process removed COD by 97%, resulting in a permeate with a residual COD level lower than 150 mg/L. Furthermore, total nitrogen, total phosphorus, total suspended solids, total solids, and conductivity were reduced by 67%, 76.2%, 94%, 96%, and 94%, respectively. Additionally, RO reduced the toxicity from 100% immobilization of Daphnia magna in the feed wastewater to zero in the permeate, while phytotoxicity was substantially reduced to values below 21%.

Overall, RO and NF technologies demonstrate critical versatility in advancing wine production through targeted applications: must concentration (achieving ~45°Brix via hybrid RO/NF systems), precise ethanol reduction (2.5–6.7% rejection rates using polyamide or cellulose acetate membranes), and recovery of high-value polyphenols (>90% retention). Membrane selection—guided by molecular weight cutoff (MWCO), hydrophilicity, and charge—must align with process objectives, balancing flux efficiency against fouling risks. Hybrid systems (e.g., RO with evaporative perstraction) and optimized operational parameters (pressure, temperature) mitigate trade-offs between selectivity and sensory integrity. For wastewater, integrated NF-RO configurations achieve >95% COD removal, enabling resource circularity.

## 4. Beer Industry

### 4.1. Alcohol-Free and Low-Alcohol Beer Production

The production of alcohol-free and low-alcohol beers has been revolutionized by membrane-based technologies, particularly RO and NF. These methods are highly effective at removing ethanol while retaining the beer’s essential flavor compounds, making them superior to traditional thermal dealcoholization techniques. Reverse osmosis involves passing beer through a semi-permeable membrane under high pressure, separating ethanol and water from larger flavor molecules. This process is widely used for its efficiency in ethanol removal, but it requires careful optimization to minimize fouling and maintain flavor integrity. Alcantara et al. [57] explored the dealcoholization of extra stout beer using RO and found that while the beer’s quality parameters (e.g., bitterness, color, and aroma) remained unchanged, the fouling rate coefficient increased over time, leading to reduced permeate flow. The reverse osmosis process effectively reduced the ethanol content of beer from an initial 4.34% *v*/*v* to values ranging between 1.45% and 2.75% *v*/*v*, depending on operating conditions. The highest ethanol reduction was achieved at a transmembrane pressure (TMP) of 30 bar, where the ethanol content decreased to 1.45% *v*/*v*. The process retained over 99% of the real extract and 100% of the bitterness compounds in retentate, ensuring minimal impact on beer quality. However, complete dealcoholizing (below 0.5% *v*/*v*) was not achieved due to the declining ethanol flux over time, requiring extended processing durations for further reduction [58].

Nanofiltration, on the other hand, operates at lower pressures than RO and selectively removes ethanol while retaining larger flavor molecules. NF membranes have smaller pore sizes compared to ultrafiltration but larger than RO membranes, making them ideal for balancing ethanol removal and flavor retention. Bóna et al. [8] demonstrated the dealcoholizing of filtered and unfiltered lager beer using polyelectrolyte multilayer nanofiltration (PEM NF) membranes. The process successfully reduced the alcohol content of filtered and unfiltered lager beer from 4.7 to 5.0% *v*/*v* to below 0.5% *v*/*v* in the final dealcoholized product. The alcohol passage through the membrane was nearly 100%, indicating high efficiency in ethanol removal. The real extract loss ranged between 15 to 18%, affecting the beer’s overall composition. Additionally, significant losses of sodium and potassium ions (~50% passage) were observed, necessitating post-treatment mineral and glycerol adjustments to enhance flavor. The study also highlights the potential of PEM NF membranes for producing non-alcoholic beer while maintaining a satisfactory sensory profile.

### 4.2. Removal of Undesirable Components

The presence of undesirable components, such as dimethyl sulfide (DMS) and aldehydes, can significantly impact the sensory quality of beer. DMS, a sulfur-containing compound, is often associated with off-flavors resembling cooked corn or vegetables, while aldehydes, such as acetaldehyde, can impart green apple-like flavors and are considered markers of incomplete fermentation. Traditional methods for removing these compounds include adsorption onto activated carbon or zeolites and the use of specific yeast strains with enhanced metabolic activity [59]. However, membrane-based technologies, particularly RO and NF, have emerged as effective alternatives for removing undesirable components while preserving the beer’s desirable flavor profile.

RO and NF membranes are increasingly being used in the beer industry to address quality issues caused by undesirable compounds. These technologies operate by selectively separating molecules based on size and charge, making them ideal for removing small, unwanted compounds like DMS and aldehydes without significantly altering the beer’s flavor [60]. NF membranes have demonstrated effectiveness in removing small, polar molecules, including aldehydes like acetaldehyde, thereby contributing to the improvement of wine sensory quality [8].

### 4.3. Innovations in Hop and Flavor Compound Retention During Dealcoholization

Several scientific studies have investigated the application of RO and NF membranes in beer dealcoholization, focusing on their effectiveness in retaining flavor compounds. RO membranes allow passage for small molecules, like ethanol, while retaining larger compounds such as sugars, proteins, and bitterness compounds. However, due to their dense structure, they may also retain some desirable flavor compounds, leading to potential modifications in the sensory profile of beer. Studies have shown that using RO at moderate pressures (e.g., 20–60 bar) can effectively reduce alcohol while maintaining hop-derived volatiles and ester compounds responsible for fruity and floral notes. Varga et al. [58] examined the dealcoholization of pale lager beer using RO at 15 ± 1 °C with an Alfa Laval RO99 membrane. The research analyzed various parameters, including ethanol content, extract content, bitterness, color, pH, turbidity, and dynamic viscosity to assess the impact of RO on beer quality. The findings suggested that appropriate operational conditions could effectively reduce alcohol content while preserving key flavor attributes. Similarly, Ramsey et al. [9] examined the impact of RO membrane technology on dealcoholizing stout and lager beers. The research revealed that RO membranes can lead to the reduction of specific volatile compounds, with significant reductions (*p* < 0.05) observed in key volatile compounds with linear structures (ethyl octanoate, octan-1-ol) compared to those with increased levels of branching (3-methyl-butyl acetate, 2-methylpropan-1-ol). This demonstrates that RO membranes can exhibit selectivity in retaining certain flavor compounds while allowing others to pass through, potentially altering the beer’s original sensory characteristics.

NF membranes, being less selective than RO, allow partial passage of ethanol while retaining larger flavor molecules. This makes them particularly attractive for partial dealcoholizing, where the alcohol content is reduced rather than completely removed. Studies indicate that NF membranes with appropriate molecular weight cutoff values (200–500 Da) can help preserve key volatiles such as isoamyl acetate, ethyl hexanoate, and linalool, which contribute to the beer’s sensory appeal. Bòna et al. [8] explored the use of hollow fiber polyelectrolyte multilayer NF membranes for beer dealcoholizing. The study found that dealcoholizing was achievable with a reasonable flux (10 L/m^2^h) at low pressures (5–8.6 bar), resulting in an acceptable-tasting beer. However, the process resulted in real extract loss of 15–18% and high inorganic salt passage, which greatly affected beer flavor. The process could be further enhanced by adding back lost salts and glycerol to improve flavors. These membrane processes offer a non-thermal approach to dealcoholizing, preserving the volatile and heat-sensitive aroma compounds that are often lost in traditional distillation or thermal evaporation methods.

### 4.4. Water Reuse and Treatment in Breweries

Water is a critical resource in the brewing process, and its efficient use and treatment are essential for sustainable brewery operations. Breweries generate significant volumes of wastewater, which can contain high levels of organic matter, nitrogen, and phosphorus [61]. Effective wastewater treatment is necessary to meet regulatory standards, reduce environmental impact, and enable water reuse. Conventional wastewater treatment methods in breweries include aerobic and anaerobic biological processes. Aerobic treatment, such as activated sludge systems, is effective in reducing organic load, while anaerobic digestion can produce biogas, offering an additional energy source [62]. However, advanced treatment technologies, such as RO and NF membranes, have gained attention for their ability to improve water quality and facilitate water reuse in breweries.

RO and NF membranes are increasingly being used in breweries to treat wastewater and enable water reuse. These technologies operate by selectively separating contaminants based on size and charge, making them highly effective for removing dissolved solids, organic compounds, and microorganisms from wastewater. RO membranes, with their smaller pore sizes, are particularly effective for desalination and the removal of trace contaminants, while NF membranes are ideal for removing larger organic molecules and multivalent ions. A notable case study by Simate et al. [63] demonstrated the use of RO membranes to treat brewery wastewater, achieving a 95% reduction in total dissolved solids (TDS) and enabling the reuse of treated water for non-potable applications such as cleaning and cooling. These studies highlight the potential of membrane technologies to address the dual challenges of wastewater treatment and water reuse in breweries. Toran et al. [64] incorporated RO in two different advanced tertiary treatment trains for brewery wastewater, highlighting its role in water treatment. In both systems, RO units were employed to enhance water polishing and improve the quality of treated water. One treatment train utilized ultrafiltration followed by RO modules, while the other employed ozonation, coagulation, microfiltration (MF), and then RO. The RO unit was designed to treat a feed flow of 4.50 m^3^/h in one system and 28 L/h in the other, with variations in operating conditions and performance observed. Notably, the RO membranes demonstrated a high rejection of salts (95%) and efficient removal of organic matter (75%) and nitrate (74%). Ultimately, the RO units proved crucial in polishing both UF and MF effluent streams to meet water quality standards suitable for potable reuse.

### 4.5. Impact on Energy Efficiency and Sustainability

The beer industry is increasingly focused on improving energy efficiency and sustainability. Dealcoholization processes, particularly those involving thermal methods, can be energy-intensive. However, recent advancements in membrane-based technologies and process integration have the potential to reduce energy consumption and enhance sustainability [62]. For instance, the use of NF and RO membranes for dealcoholization requires lower energy input compared to thermal methods, as it operates at ambient temperatures. Additionally, the recovery and reuse of water and by-products, such as spent grains and yeast, can contribute to a more sustainable brewing process [63].

Sustainability is further enhanced through the integration of RO and NF membranes with water reuse systems and wastewater treatment processes. Breweries generate significant volumes of wastewater, which can be treated using membrane technologies to recover water for reuse in brewing or cleaning processes. For instance, (NF) membranes are being used to treat spent process water, enabling the recovery of valuable by-products like sugars and proteins while ensuring water reuse [65]. Additionally, (RO) systems are employed to treat brewery wastewater, achieving high-quality effluent that meets regulatory standards and reduces freshwater consumption [66]. These applications not only conserve water resources but also minimize waste, contributing to a circular economy in the brewing industry. Sánchez et al. [67] compared a hybrid nanofiltration/distillation process for dealcoholizing beer with a standalone process. For an annual production of 720,000 L of alcohol-free beer, the total cost of the standalone variant was USD 193,600/year, while the hybrid process cost USD 205,500/year, which is 6.2% higher. However, a life cycle assessment indicated that the hybrid process has a lower environmental load. As membrane technologies continue to evolve, their role in promoting energy efficiency and sustainability in beer production is expected to grow, helping breweries meet both environmental and economic objectives.

Overall, the strategic integration of RO and NF in brewing hinges on targeted application objectives: RO achieves superior ethanol removal (1.45–2.75% *v*/*v*) and high-purity water reuse via tight molecular sieving (< 200 Da MWCO) under high pressure (20–60 bar), albeit with fouling trade-offs and incomplete dealcoholization, while NF enables sub-0.5% *v*/*v* ethanol reduction at lower pressures (5–8.6 bar) via charge/size selectivity but risks extract loss (15–18%) and ion depletion, necessitating post-treatment. Membrane selection must prioritize MWCO, hydrophobicity, and fouling resistance to balance flavor fidelity (retention of isoamyl acetate, linalool), regulatory compliance, and process scalability, with RO favored for rigorous contaminant rejection and NF for partial dealcoholization with minimized thermal degradation of volatiles.

## 5. Challenges and Limitations of RO and NF in the Industry

RO and NF are widely used in the wine and beer industry for concentration, dealcoholization, aroma recovery, recovery of valuable by-products, and wastewater treatment processes. However, their application is not without challenges and limitations. Key issues include membrane fouling, energy consumption, sensory quality impacts, and regulatory considerations. These factors must be carefully managed to ensure the economic viability and product quality of the final beverage.

### 5.1. Membrane Fouling and Cleaning Requirements

Membrane fouling is a significant challenge in RO and NF processes, particularly in the wine and beer industry, where the feed streams contain complex mixtures of organic compounds, such as polysaccharides, proteins, and polyphenols. These compounds can adsorb onto the membrane surface or within its pores, leading to reduced flux, increased transmembrane pressure, and decreased separation efficiency [14]. Fouling not only affects process efficiency but also increases operational costs due to the need for frequent cleaning and membrane replacement. Cleaning protocols often involve chemical agents such as sodium hydroxide, citric acid, or enzymatic cleaners, which can degrade membrane materials over time. Catarino et al. [66] highlighted that fouling in wine dealcoholization processes significantly reduced membrane lifespan, necessitating rigorous cleaning cycles. Despite advances in fouling-resistant membranes, fouling remains a persistent issue that requires ongoing research and optimization.

### 5.2. Energy Consumption and Operational Costs

RO and NF are energy-intensive processes due to the high pressures required to overcome osmotic pressure and achieve separation. In the wine and beer industry, where large volumes of liquid are processed, energy consumption can be a major operational cost. For instance, dealcoholization of beer using RO typically requires pressures of 20–40 bar, while wine concentration can demand even higher pressures [66]. The energy requirement is further exacerbated by fouling, which increases resistance to flow. Additionally, the need for pre-treatment steps, such as microfiltration or ultrafiltration, to remove particulate matter adds to the overall energy burden. Labanda et al. [68] demonstrated that the energy costs associated with RO-based wine dealcoholization accounted for nearly 30% of the total operational expenses. While membrane processes such as RO and NF can offer energy savings compared to thermal methods by avoiding high-temperature operations, their reliance on high-pressure pumps means that electrical energy consumption remains substantial. As such, energy use continues to be a critical factor limiting their widespread adoption, particularly in large-scale beverage processing.

### 5.3. Impact on Sensory Quality and Product Stability

The sensory quality of wine and beer is a critical factor in consumer acceptance, and RO and NF processes can significantly influence flavor, aroma, and mouthfeel. Dealcoholization using RO can lead to the loss of volatile aroma compounds, which are essential for the characteristic flavor profile of wine and beer [3,4,8]. Similarly, the removal of polyphenols during NF can alter the color and astringency of wine, affecting its sensory attributes. Diban et al. [69] found that NF-treated wines exhibited reduced complexity and intensity in aroma compared to untreated wines. Furthermore, the stability of the final product can be compromised if key components, such as proteins or polyphenols, are removed during filtration. This necessitates careful optimization of process parameters to balance alcohol reduction or concentration with sensory quality preservation.

### 5.4. Regulatory Considerations and Consumer Acceptance

Regulatory frameworks and consumer perceptions play a significant role in the adoption of RO and NF technologies in the wine and beer industry. In many regions, dealcoholized or low-alcohol beverages must meet specific legal standards to be marketed as such. The European union (EU) recently created categories for products to be marketed under the legal names “dealcoholized wine” and “partially dealcoholized wine”, defined as having actual alcoholic strengths of “no more than 0.5% *v*/*v* ethanol” and “above 0.5% *v*/*v* ethanol and below the minimum actual alcoholic strength of the wine category”, respectively [70,71,72,73]. Compliance with these regulations often requires precise control over the RO or NF process, which can be challenging. Additionally, consumer acceptance of dealcoholized or membrane-treated beverages varies widely. While some consumers appreciate the health benefits of low-alcohol options, others perceive them as inferior in quality. Meillon et al. [73] found that consumers preferred traditionally brewed beers over dealcoholized versions, citing differences in flavor and mouthfeel. This highlights the need for clear communication and marketing strategies to address consumer concerns.

## 6. Conclusions

RO and NF membrane processes have demonstrated substantial potential in addressing critical challenges within the wine and beer industries, including alcohol modulation, sugar adjustment in musts, recovery of bioactive compounds, and sustainable water management. These technologies leverage size-exclusion and charge-based separation mechanisms to achieve selective solute retention or permeation, offering advantages over conventional thermal and chemical methods by minimizing thermal degradation and preserving organoleptic integrity. In winemaking, RO enables the selective removal of ethanol and water, supporting must concentration and low-alcohol wine production when combined with appropriate reconstruction techniques. NF, in turn, facilitates polyphenol recovery from grape pomace, aligning with circular economy principles. In brewing, NF supports ethanol reduction without compromising hop-derived flavor compounds, and RO enhances water reuse efficiency, critical in water-stressed regions.

Despite their efficacy, challenges such as membrane fouling due to polysaccharides, phenolics, or colloidal aggregates, and trade-offs between permeate flux and selectivity, underscore the need for optimized membrane materials and process configurations. Additionally, energy consumption, though lower than thermal alternatives, remains a barrier to scalability, necessitating advances in low-pressure membranes and renewable energy integration. Sensory studies reveal that while RO/NF minimally alters volatile aroma profiles compared to traditional methods, subtle shifts in acidity or bitterness require rigorous process calibration. Regulatory frameworks must evolve to standardize labeling for membrane-processed beverages and validate their safety and quality claims.

Future research should prioritize the development of fouling-resistant, high-selectivity membranes tailored to complex beverage matrices, alongside hybrid systems integrating RO/NF with electrodialysis for enhanced resource recovery. Longitudinal studies on membrane-treated product stability, coupled with metabolomic fingerprinting to quantify sensory impacts, will bridge existing knowledge gaps. Collaborative efforts among material scientists, microbiologists, and industry stakeholders are essential to harmonize technological innovation with traditional practices, ensuring RO/NF adoption enhances sustainability without compromising the cultural and sensory heritage of wine and beer. By addressing these interdisciplinary challenges, membrane processes can transition from niche applications to cornerstone technologies in the evolving landscape of beverage production.

## Figures and Tables

**Table 1:** Characteristics of membrane processes used in wine and beer industry and principal changes in wine and beer compositions [2,8,9,14].

Membrane Process	Driving Force	Pressure Range	Pore Diameter	Membrane Material	Principle Changes in Wine Composition	Principle Changes in Beer Composition
NF	Pressure gradient	2–40 bar	0.001–0.005 μm	Asymmetrical polymeric or thin-film composite membranes	Retains sugars, some organic acids, monomeric phenolic compounds. Increases anthocyanins and resveratrol in red wine, variable effects on phenolic compounds. Minor loss of aroma compounds in white wines. Decrease in astringency in some red wines.	Retains compounds with high molecular weight, such as sugars, flavor compounds, and colors. Loss of low-molecular compounds, such as water, alcohol, and mono-ion compounds and salts altering taste. Retention of aroma and color compounds. Loss of CO_2_ during process.
RO	Pressure gradient	10–100 bar	<0.001 μm	Thin-film or dense composite or polymeric membranes	Retains sugars, some organic acids, monomeric phenolic and ions. Stable phenolic index and anthocyanin content. Enhances color intensity. Decreases mouthfeel and complexity in some wines. Increases astringency.	Retains compounds with high-molecular compounds, such as sugars, pigments, flavors, and bitter compounds. Losses of water, alcohol. Retains aroma and taste.

## Data Availability

No new data were created or analyzed in this study. Data sharing is not applicable to this article.

## References

[1] Chan S.M., Adzahan N.M., Ab Karim M.S., Karim R., Lasekan O., Regenstein J.M. (2012). Consumer Preferences and Perceptions on Dealcoholised Wine. J. Food Prod. Mark..

[2] Silva P. (2024). Low-Alcohol and Nonalcoholic Wines: From Production to Cardiovascular Health, along with Their Economic Effects. Beverages.

[3] Kumar Y., Ricci A., Parpinello G.P., Versari A. (2024). Dealcoholized Wine: A Scoping Review of Volatile and Non-Volatile Profiles, Consumer Perception, and Health Benefits. Food Bioprocess Technol..

[4] Sam F.E., Ma T.-Z., Salifu R., Wang J., Jiang Y.-M., Zhang B., Han S.-Y. (2021). Techniques for Dealcoholization of Wines: Their Impact on Wine Phenolic Composition, Volatile Composition, and Sensory Characteristics. Foods.

[5] Teow Y.H., Sum J.Y., Ho K.C., Mohammad A.W. (2021). Principles of Nanofiltration Membrane Processes. Osmosis Engineering.

[6] Shenvi S.S., Isloor A.M., Ismail A.F. (2015). A Review on RO Membrane Technology: Developments and Challenges. Desalination.

[7] Kumar Y., Cassano A., Conidi C., Gottardi D., Ricci A., Parpinello G.P., Versari A. (2025). Evaluation of Physicochemical Characteristics, Color and Volatile Profile of Low Alcohol Beverage Based on Concentrated White Wine Produced by NF and RO Membranes. Sep. Purif. Technol..

[8] Bóna Á., Varga Á., Galambos I., Nemestóthy N. (2023). Dealcoholization of Unfiltered and Filtered Lager Beer by Hollow Fiber Polyelectrolyte Multilayer Nanofiltration Membranes—The Effect of Ion Rejection. Membranes.

[9] Ramsey I., Yang Q., Fisk I., Ayed C., Ford R. (2021). Assessing the Sensory and Physicochemical Impact of Reverse Osmosis Membrane Tech-nology to Dealcoholize Two Different Beer Styles. Food Chem. X.

[10] Yang Z., Zhou Y., Feng Z., Rui X., Zhang T., Zhang Z. (2019). A Review on Reverse Osmosis and Nanofiltration Membranes for Water Purification. Polymers.

[11] Sam F.E., Ma T., Liang Y., Qiang W., Atuna R.A., Amagloh F.K., Morata A., Han S. (2021). Comparison between Membrane and Thermal Dealcoholization Methods: Their Impact on the Chemical Parameters, Volatile Composition, and Sensory Characteristics of Wines. Membranes.

[12] García-Martín N., Perez-Magariño S., Ortega-Heras M., González-Huerta C., Mihnea M., González-Sanjosé M.L., Palacio L., Prádanos P., Hernández A. (2010). Sugar Reduction in Musts with Nanofiltration Membranes to Obtain Low Alcohol-Content Wines. Sep. Purif. Technol..

[13] García-Martín N., Perez-Magariño S., Ortega-Heras M., González-Huerta C., Mihnea M., González-Sanjosé M.L., Palacio L., Prádanos P., Hernández A. (2011). Sugar Reduction in White and Red Musts with Nanofiltration Membranes. Desalination Water Treat..

[14] El Rayess Y., Castro-Muñoz R., Cassano A. (2024). Current Advances in Membrane Processing of Wines: A Comprehensive Review. Trends Food Sci. Technol..

[15] Ivić I., Kopjar M., Obhođaš J., Vinković A., Pichler D., Mesić J., Pichler A. (2021). Concentration with Nanofiltration of Red Wine Cabernet Sauvignon Produced from Conventionally and Ecologically Grown Grapes: Effect on Volatile Compounds and Chemical Composition. Membranes.

[16] Giacobbo A., Moura Bernardes A., Filipe Rosa M., De Pinho M. (2018). Concentration Polarization in Ultrafiltration/Nanofiltration for the Recovery of Polyphenols from Winery Wastewaters. Membranes.

[17] Oro C.E.D., Puton B.M.S., Venquiaruto L.D., Dallago R.M., Arend G.D., Tres M.V. (2025). The Role of Membranes in Modern Winemaking: From Clarification to Dealcoholization. Membranes.

[18] Genova J., Dencheva-Zarkova M., Yankov D., Tsibranska I. (2024). Influence of Transmembrane Pressure on the Retention of Bioactive Compounds and Alcohol Content in Red Wine Nanofiltration. WSEAS Trans. Biol. Biomed..

[19] Artiga P., Carballa M., Garrido J.M., Méndez R. (2007). Treatment of Winery Wastewaters in a Membrane Submerged Bioreactor. Water Sci. Technol..

[20] Tapia-Quirós P., Montenegro-Landívar M.F., Reig M., Vecino X., Saurina J., Granados M., Cortina J.L. (2022). Integration of Nanofiltration and Reverse Osmosis Technologies in Polyphenols Recovery Schemes from Winery and Olive Mill Wastes by Aqueous-Based Processing. Membranes.

[21] López-Borrell A., López-Pérez M.-F., Cardona S.C., Lora-García J. (2022). Experimental Study and Mathematical Modeling of a Nanofiltration Membrane System for the Recovery of Polyphenols from Wine Lees. Membranes.

[22] Salanță L.C., Coldea T.E., Ignat M.V., Pop C.R., Tofană M., Mudura E., Borșa A., Pasqualone A., Anjos O., Zhao H. (2020). Functionality of Special Beer Processes and Potential Health Benefits. Processes.

[23] Nehra M. (2022). Non Alcoholic Beers: Review and Methods. Madridge J. Food Technol..

[24] Maeda Y. (2024). Fouling of Reverse Osmosis (RO) and Nanofiltration (NF) Membranes by Low Molecular Weight Organic Compounds (LMWOCs), Part 1: Fundamentals and Mechanism. Membranes.

[25] Ahmed M.A., Amin S., Mohamed A.A. (2023). Fouling in Reverse Osmosis Membranes: Monitoring, Characterization, Mitigation Strategies and Future Directions. Heliyon.

[26] Balcik C. (2021). Understanding the Operational Problems and Fouling Characterization of RO Membrane Used for Brackish Water Treatment via Membrane Autopsy. Water Sci. Technol..

[27] Charcosset C., Basile A., Charcosset C. (2016). Ultrafiltration, Microfiltration, Nanofiltration and Reverse Osmosis in Integrated Membrane Processes. Integrated Membrane Systems and Processes.

[28] Charcosset C. (2021). Classical and Recent Applications of Membrane Processes in the Food Industry. Food Eng. Rev..

[29] Kumar Y., Cassano A., Conidi C., Ricci A., Parpinello G.P., Versari A. (2024). Evaluating Membrane Behavior to Ethanol-Water Mixtures and Wine: A Comparative Investigation. LWT.

[30] Destani F., Naccarato A., Tagarelli A., Cassano A. (2020). Recovery of Aromatics from Orange Juice Evaporator Condensate Streams by Reverse Osmosis. Membranes.

[31] Ivić I., Kopjar M., Jukić V., Bošnjak M., Maglica M., Mesić J., Pichler A. (2021). Aroma Profile and Chemical Composition of Reverse Osmosis and Nanofiltration Concentrates of Red Wine Cabernet Sauvignon. Molecules.

[32] Choi J.-S., Hwang T.-M., Lee S., Hong S. (2009). A Systematic Approach to Determine the Fouling Index for a RO/NF Membrane Process. Desalination.

[33] Banvolgyi S., Savaş Bahçeci K., Vatai G., Bekassy S., Bekassy-Molnar E. (2016). Partial Dealcoholization of Red Wine by Nanofiltration and Its Effect on Anthocyanin and Resveratrol Levels. Food Sci. Technol. Int..

[34] Bianchi B., Molino B., Catalano F., Giametta F., Molino A.J., Ambrosone L. (2023). A Novel Approach to Optimize the Industrial Process of Membrane Concentration of Grape Musts. ChemEngineering.

[35] Gurak P.D., Cabral L.M.C., Rocha-Leão M.H.M., Matta V.M., Freitas S.P. (2010). Quality Evaluation of Grape Juice Concentrated by Reverse Osmosis. J. Food Eng..

[36] Drioli E., Fontananova E. (2004). Membrane Technology and Sustainable Growth. Chem. Eng. Res. Des..

[37] Pati S., La Notte D., Clodoveo M.L., Cicco G., Esti M. (2014). Reverse Osmosis and Nanofiltration Membranes for the Improvement of Must Quality. Eur. Food Res. Technol..

[38] Rosa Santos F., Catarino I., Geraldes V., Pinho M.N. (2008). Concentration and Rectification of Grape Must by Nanofiltration. Am. J. Enol. Vitic..

[39] Versari A., Ferrarini R., Parpinello G.P., Galassi S. (2003). Concentration of Grape Must by Nanofiltration Membranes. Food Bioprod. Process..

[40] Kiss I., Vatai G., Bekassy-Molnar E. (2004). Must Concentrate Using Membrane Technology. Desalination.

[41] Mihnea M., González-SanJosé M.L., Ortega-Heras M., Pérez-Magariño S., García-Martin N., Palacio L., Prádanos P., Hernández A. (2012). Impact of Must Sugar Reduction by Membrane Applications on Volatile Composition of Verdejo Wines. J. Agric. Food Chem..

[42] Kumar Y., Cassano A., Conidi C., Gottardi D., Ricci A., Parpinello G.P., Versari A. (2025). White Wine Dealcoholization by Osmotic Distillation: An Experimental Study and Impact on Key Quality Parameters. J. Food Eng..

[43] Italiano L., Kumar Y., Matthias S., Christmann M. Evaluation of Dialysis Membrane Efficiency in Wine Dealcoholisation Process. Proceedings of the 45th World Congress of Vine and Wine (OIV 2024).

[44] Catarino M., Mendes A. (2011). Dealcoholizing Wine by Membrane Separation Processes. Innov. Food Sci. Emerg. Technol..

[45] Gil M., Estévez S., Kontoudakis N., Fort F., Canals J.M., Zamora F. (2013). Influence of Partial Dealcoholization by Reverse Osmosis on Red Wine Composition and Sensory Characteristics. Eur. Food Res. Technol..

[46] Pham D.-T., Ristic R., Stockdale V.J., Jeffery D.W., Tuke J., Wilkinson K. (2020). Influence of Partial Dealcoholization on the Composition and Sensory Properties of Cabernet Sauvignon Wines. Food Chem..

[47] Peng P., Lan Y., Liang L., Jia K. (2021). Membranes for Bioethanol Production by Pervaporation. Biotechnol. Biofuels.

[48] Verhoef A., Figoli A., Leen B., Bettens B., Drioli E., Van Der Bruggen B. (2008). Performance of a Nanofiltration Membrane for Removal of Ethanol from Aqueous Solutions by Pervaporation. Sep. Purif. Technol..

[49] Thai P.T.N., Pham X.M., Nguyen T.B., Le T.M., Viet Tran C.B., Phong M.T., Tran L.-H. (2021). Preparation and Characterization of PVA Thin-Film Composite Membrane for Pervaporation Dehydration of Ethanol Solution. IOP Conf. Ser. Earth Environ. Sci..

[50] Kontogiannopoulos K.N., Patsios S.I., Mitrouli S.T., Karabelas A.J. (2017). Tartaric Acid and Polyphenols Recovery from Winery Waste Lees Using Membrane Separation Processes: Tartaric Acid and Polyphenols Recovery from Winery Waste Lees. J. Chem. Technol. Biotechnol..

[51] Bolzonella D., Papa M., Da Ros C., Anga Muthukumar L., Rosso D. (2019). Winery Wastewater Treatment: A Critical Overview of Advanced Biological Processes. Crit. Rev. Biotechnol..

[52] Bolzonella D., Fatone F., Pavan P., Cecchi F. (2010). Application of a Membrane Bioreactor for Winery Wastewater Treatment. Water Sci. Technol..

[53] Nataraj S.K., Hosamani K.M., Aminabhavi T.M. (2006). Distillery Wastewater Treatment by the Membrane-Based Nanofiltration and Reverse Osmosis Processes. Water Res..

[54] Víctor-Ortega M.D., Martins R.C., Gando-Ferreira L.M., Quinta-Ferreira R.M. (2017). Recovery of Phenolic Compounds from Wastewaters Through Micellar Enhanced Ultrafiltration. Colloids Surf. Physicochem. Eng. Asp..

[55] Santos J.R.F., Rodrigues R.P., Quina M.J., Gando-Ferreira L.M. (2023). Recovery of Value-Added Compounds from Winery Wastewater: A Review and Bibliometric Analysis. Water.

[56] Ioannou L.A., Michael C., Vakondios N., Drosou K., Xekoukoulotakis N.P., Diamadopoulos E., Fatta-Kassinos D. (2013). Winery Wastewater Purification by Reverse Osmosis and Oxidation of the Concentrate by Solar Photo-FENTON. Sep. Purif. Technol..

[57] Alcantara B.M., Marques D.R., Chinellato M.M., Marchi L.B., Costa S.C., Monteiro A.R.G. (2016). Assessment of Quality and Production Process of a Non-Alcoholic Stout Beer Using Reverse Osmosis. J. Inst. Brew..

[58] Varga Á., Bihari-Lucena E., Ladányi M., Szabó-Nótin B., Galambos I., Koris A. (2023). Experimental Study and Modeling of Beer Dealcoholization via Reverse Osmosis. Membranes.

[59] Krogerus K., Gibson B.R. (2013). 125th Anniversary Review: Diacetyl and Its Control During Brewery Fermentation: Diacetyl and Its Control During Brewery Fermentation. J. Inst. Brew..

[60] Kebede T.B. (2018). Waste Water Treatment in Brewery Industry, Review. Int. J. Eng. Dev. Res..

[61] Fillaudeau L., Blanpain-Avet P., Daufin G. (2006). Water, Wastewater and Waste Management in Brewing Industries. J. Clean. Prod..

[62] Simate G.S., Cluett J., Iyuke S.E., Musapatika E.T., Ndlovu S., Walubita L.F., Alvarez A.E. (2011). The Treatment of Brewery Wastewater for Reuse: State of the Art. Desalination.

[63] Toran M.S., Labrador P.F., Ciriza J.F., Asensio Y., Reigersman A., Arevalo J., Rogalla F., Monsalvo V.M. (2021). Membrane-Based Processes to Obtain High-Quality Water From Brewery Wastewater. Front. Chem. Eng..

[64] Muller C., Neves L.E., Gomes L., Guimarães M., Ghesti G. (2020). Processes for Alcohol-Free Beer Production: A Review. Food Sci. Technol..

[65] Catarino M., Mendes A., Madeira L.M., Ferreira A. (2007). Alcohol Removal From Beer by Reverse Osmosis. Sep. Sci. Technol..

[66] Sánchez R.J., Figueroa Paredes D.A., Laoretani D.S., Villada Y., Fuentes M., Espinosa J. (2021). On the Conceptual Design of the Hybrid Nanofiltration/Distillation Process in the Production of Alcohol-Free Beers. Sep. Purif. Technol..

[67] Labanda J., Vichi S., Llorens J., López-Tamames E. (2009). Membrane Separation Technology for the Reduction of Alcoholic Degree of a White Model Wine. LWT Food Sci. Technol..

[68] Diban N., Arruti A., Barceló A., Puxeu M., Urtiaga A., Ortiz I. (2013). Membrane Dealcoholization of Different Wine Varieties Reducing Aroma Losses. Modeling and Experimental Validation. Innov. Food Sci. Emerg. Technol..

[69] OIV (2021). New CAP Gives Official Status to ‘De-Alcoholised Wines’ According to the OIV’s Adopted Framework.

[70] European Parliament, Council of the European Union (2021). EU Regulation 2117/2021. Establishing a Common Organisation of the Markets in Agricultural Products, (EU) No 1151/2012 on Quality Schemes for Agricultural Products and Foodstuffs, (EU) No 251/2014 on the Definition, De-scription, Presentation, Labelling and the Protection of Geographical Indications of Aromatised Wine Products and (EU) No 228/2013 Laying down Specific Measures for Agriculture in the Outermost Regions of the Union. Off. J. Eur. Union L.

[71] Kumar Y., Ricci A., Wang G., Parpinello G.P., Versari A. (2024). The Effect of Ethanol, SO_2_, and Transition Metals on Browning Kinetics in Low- and No-Alcohol Model Wine. J. Food Biochem..

[72] Kumar Y., Italiano L., Schmitt M., Ricci A., Parpinello G.P., Versari A. Exploring Changes in Browning Kinetics, Color, and Antioxidants Due to Dealcoholization of Wine. Proceedings of the 45th World Congress of Vine and Wine (OIV 2024).

[73] Meillon S., Urbano C., Guillot G., Schlich P. (2010). Acceptability of Partially Dealcoholized Wines—Measuring the Impact of Sensory and Information Cues on Overall Liking in Real-Life Settings. Food Qual. Prefer..

